# Acute calcareous corneal degeneration in a patient with chronic graft-versus-host disease

**DOI:** 10.22336/rjo.2024.10

**Published:** 2024

**Authors:** Marta Gema Solaz Ruiz, Lorena Azorín Pérez, Carlos Cauto Picazo, Laura Sánchez Sanz, Ana Hervás Ontiveros, Enrique España Gregori

**Affiliations:** * La Fe University and Polytechnic Hospital, Valencia, Spain; ** Lluis Alcanyis Hospital, Valencia, Spain

**Keywords:** chronic graft-versus-host disease, calcium deposition, calcareous corneal degeneration, keratoconjunctivitis sicca, corneal perforation

## Abstract

**Objective:** To describe acute calcareous corneal degeneration as a complication of chronic graft-versus-host disease.

**Materials and methods:** Clinical case and review of the literature.

**Results:** We presented a case of bilateral acute calcareous corneal degeneration in a patient with chronic graft-versus-host disease.

**Conclusions:** Chronic graft-versus-host disease (cGVHD) occurs in 50-70% of bone marrow transplantation patients, the most frequent ocular complication being keratoconjunctivitis sicca (KCS). Calcareous corneal degeneration is a type of calcium deposition that can be secondary to chronic ocular inflammation or dry eye, but there are few cases reported of acute calcareous corneal degeneration and recurrent perforation in cGVHD.

**Abbreviations:** GVHD = Chronic graft-versus-host disease, aGVHD = Acute graft-versus-host disease, cGVHD = Chronic graft-versus-host disease, KCS = Keratoconjunctivitis sicca, PKP = Penetrating keratoplasty, AMT = Amniotic membrane transplantation, PRGF = Plasma rich in growth factors, OD = Right eye, OS = Left eye

## Introduction

Graft-versus-host disease (GVHD) is a significant complication in patients following bone marrow transplantation (BMT).

Keratoconjunctivitis sicca (KCS) is the most prevalent complication in the eyes, resulting from reduced tear production and changes in the composition of the tear film. Other ocular complications include cicatricial lagophthalmos, persistent corneal epithelial defect, corneal ulcers, and corneal melting [**1**,**2**].

The deposition of calcium in the cornea can be categorized as either calcific band keratopathy or calcareous corneal degeneration [**3**]. These conditions have been observed in individuals with chronic ocular inflammation, dry eye, or after undergoing ocular surgical procedures. On the other hand, instances of acute calcareous corneal degeneration are infrequently reported in individuals with GVHD [**4**].

In this article, we presented a case involving recurrent corneal perforation and calcareous degeneration attributed to chronic graft-versus-host disease (cGVHD).

## Materials and methods

Clinical case and review of the literature. 

## Results

A 62-year-old woman diagnosed with angioimmunoblastic-type non-Hodgkin lymphoma in May 2021 underwent an allogeneic hematopoietic stem cell transplant in March 2022. In the days following the procedure, she developed acute graft-versus-host disease (aGVHD) with cutaneous and intestinal complications. She was treated with corticosteroids and immunosuppressants.

After ten months of post-allogeneic hematopoietic stem cell transplant, she manifested cGVHD characterized by cutaneous and ocular complications. This condition was managed with a first-line treatment including corticosteroids, ruxolitinib, sirolimus, and phototherapy. Due to the limited response to the initial treatment, a second-line approach was necessary, incorporating mycophenolate mofetil, ruxolitinib, and sirolimus. A third line of treatment was required, with the addition of extracorporeal photopheresis. The cutaneous symptoms improved; however, the ocular symptoms did not show the same improvement. The patient developed a severe KCS with persistent corneal erosions and corneal thinning.

The ocular management initially involved intense eye lubrication using artificial tears, insulin eye drops, and lubricating ointments, given the severe dry eye symptoms. Additionally, topical antibiotics were administered for recurrent epithelial erosions.

One month after the onset of cGVHD, a decision was made to perform a penetrating keratoplasty (PK) due to ocular perforation in the left eye (OS). In the right eye (OD), a plasma rich in growth factors (PRGF) membrane was placed due to the presence of ulcers with severe corneal thinning.

One month after the PK in the OS, an emergency evisceration was performed on this eye due to a significant ocular perforation. In the OD, topical ciclosporin and amniotic membrane transplantation (AMT) were added to the treatment due to the start of severe calcium corneal degeneration.

**Fig. 1:**
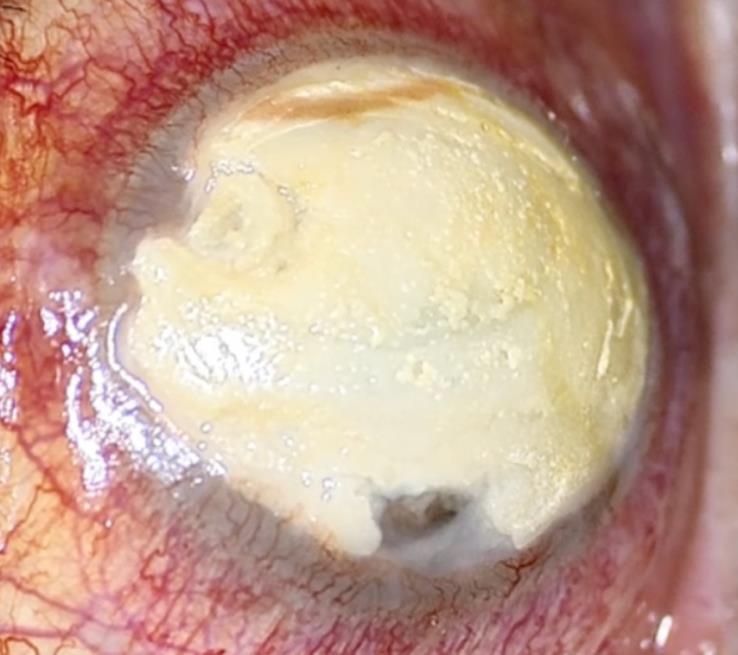
Acute calcareous corneal degeneration in the OD

**Fig. 2:**
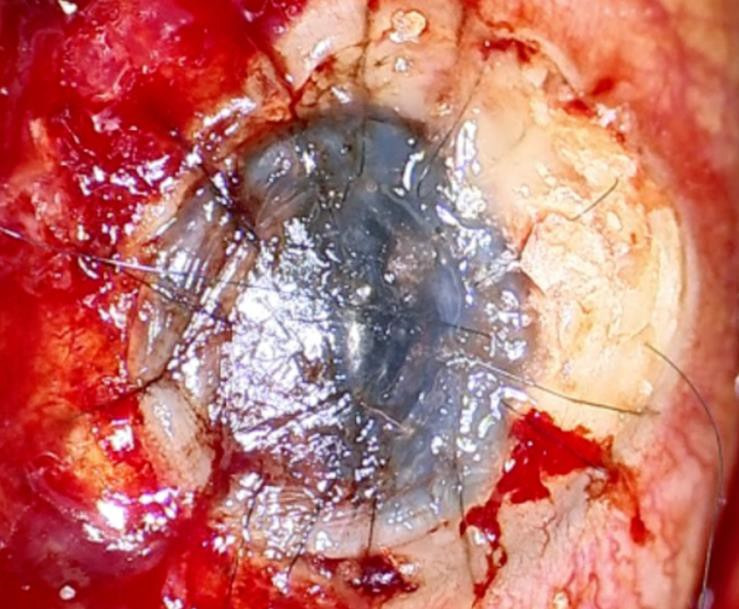
Result of the penetrating keratoplasty in the OD

Months later, our patient experienced a worsening of her ocular condition, developing acute calcareous corneal degeneration (ACCD) characterized by calcium plaques across the entire diameter of her cornea and the presence of corneal microperforations in her OD (**Fig. 1**). The decision was made to perform a PK (**Fig. 2**). However, two weeks later, she presented with new microperforations over the donor corneal button due to the recurrence of calcium plaques in the same area. It was decided to preserve the eye, choosing to use cyanoacrylate tissue adhesive. The procedure was not successful, due to the recurrence of extensive corneal perforation, and the final therapeutic alternative considered was evisceration of the OD.

## Discussion

ACCD is a rare ocular condition characterized by the deposition of calcium in all the corneal layers [**4**]. This condition can manifest as either metastatic or dystrophic. Metastatic calcification is associated with abnormal levels of serum calcium or phosphate. On the other hand, dystrophic calcification occurs as a result of localized tissue damage in cases of chronic ocular inflammation. This can be observed in eyes with a history of trauma, persistent corneal ulcers, or following surgical procedures [**5**]. 

GVHD is a complication that can occur after allogeneic hematopoietic stem cell transplantation. The immune cells recognize host tissues as foreign and initiate an inflammatory cascade. This inflammation can disrupt the normal homeostasis of the cornea [**6**]. The exact mechanisms leading to ACCD in the context of GVHD are not fully understood, it may result from a combination of factors, including altered tear film composition, chronic inflammation, and impaired corneal epithelial function.

Managing ACCD in the context of GVHD requires a multidisciplinary approach. Ocular treatment includes a combination of medical and surgical procedures tailored to the individual patient’s needs.

Penetrating keratoplasty (PKP) can preserve the integrity of the eye in patients with large corneal perforations [**7**]. In more challenging cases, employing aggressive interventions such as amniotic membrane transplantation (AMT), topical autologous serum, cyanoacrylate glue, tarsorrhaphy, and a combination of these techniques, may contribute to vision preservation [**8**]. 

Amniotic membrane (AM) has anti-inflammatory, anti-microbial, anti-angiogenic, and low immunogenicity properties. It stimulates the process of corneal epithelialization and promotes the differentiation of the epithelium. Additionally, it enhances the adhesion of basal epithelial cells and acts as a preventive measure against epithelial cell apoptosis. AMT could be a good solution for patients with persistent corneal epithelial defects [**9**]. However, in our patient, it had a limited effect.

Topical autologous serum has a composition similar to tears produced by the lacrimal gland, incorporating various components such as epithelial growth factor, vitamin A, and others. These constituents may contribute to a positive impact on the corneal epithelium [**10**]. Nevertheless, the application of autologous serum showed restricted efficacy in facilitating the re-establishment of the corneal epithelium in this case.

The utility of cyanoacrylate glue in addressing corneal perforations and descemetoceles is well-documented. It plays a role in halting stromal melting and serves as a barrier to both the tear film and the early regenerating epithelium. Additionally, it has notable bacteriostatic activity against Gram-positive organisms [**11**]. However, the use of it in our patient demonstrated limited effectiveness, since the cornea perforated again.

Tarsorrhaphy is a surgical procedure involving the closure of the eyelids, aimed at reducing the width of the palpebral fissure. This surgical approach proves effective in promoting corneal re-epithelialization and providing symptomatic relief for individuals with persistent corneal epithelial defects. A recent study has shown that tarsorrhaphy with a sutureless amniotic membrane expedites the healing process of the ocular surface and improves the effectiveness of the amniotic membrane [**12**]. 

## Conclusion

To conclude, the relationship between ACCD and GVHD underscores the complex interplay between systemic immune responses and ocular manifestations. Further research is needed to elucidate the specific mechanisms leading to ACCD in the context of GVHD and to develop targeted therapeutic strategies for affected individuals.

**Conflict of Interest Statement**

The authors declare no conflict of interest.

**Informed Consent and Human and Animal Rights Statement**

Informed consent has been obtained from all individuals included in this study. Patient consent to publish the case and images were gathered.

**Authorization for the use of human subjects**

Ethical approval: The research related to human use complies with all the relevant national regulations, and institutional policies, as per the tenets of the Helsinki Declaration, and has been approved by the review board of La Fe University and Polytechnic Hospital, Valencia, Spain.

**Acknowledgments**

None.

**Sources of Funding**

The authors received no financial support for the research, authorship, and/or publication of this article.

**Disclosures**

None.
